# Chromosome-level assemblies of cultivated water chestnut *Trapa bicornis* and its wild relative *Trapa incisa*

**DOI:** 10.1038/s41597-023-02270-4

**Published:** 2023-06-24

**Authors:** Minghao Qu, Xiangrong Fan, Chenlu Hao, Yi Zheng, Sumin Guo, Sen Wang, Wei Li, Yanqin Xu, Lei Gao, Yuanyuan Chen

**Affiliations:** 1 Key Laboratory of Plant Germplasm Enhancement and Specialty Agriculture, Plant Germplasm Research Center, Wuhan Botanical Garden, Innovative Academy of Seed Design, Chinese Academy of Sciences, Wuhan, Hubei 430074 China; 2 University of Chinese Academy of Sciences, Beijing, 100049 China; 3 Aquatic Plant Research Center, Wuhan Botanical Garden, Chinese Academy of Sciences, Wuhan, Hubei 430074 China; 4 Hubei Key laboratory of Wetland evolution & ecological restoration, Wuhan Botanical Garden, Chinese academy of sciences, Wuhan, Hubei 430074 China; 5 Research Center for Ecology, College of Science, Tibet University, Lhasa, Tibet 850000 China; 6 Beijing Key Laboratory for Agricultural Application and New Technique, College of Plant Science and Technology, Bioinformatics Center, Beijing University of Agriculture, Beijing, 102206 China; 7 College of Pharmacy, Jiangxi University of Chinese Medicine, Nanchang, Jiangxi 330004 China; 8 Hubei Hongshan Laboratory, Wuhan, Hubei 430070 China

**Keywords:** Plant genetics, Chromosomes

## Abstract

Water chestnut (*Trapa* L.) is a floating-leaved aquatic plant with high edible and medicinal value. In this study, we presented chromosome-level genome assemblies of cultivated large-seed species *Trapa bicornis* and its wild small-seed relative *Trapa incisa* by using PacBio HiFi long reads and Hi-C technology. The *T. bicornis* and *T. incisa* assemblies consisted of 479.90 Mb and 463.97 Mb contigs with N50 values of 13.52 Mb and 13.77 Mb, respectively, and repeat contents of 62.88% and 62.49%, respectively. A total of 33,306 and 33,315 protein-coding genes were predicted in *T. bicornis* and *T. incisa* assemblies, respectively. There were 159,232 structural variants affecting more than 11 thousand genes detected between the two genomes. The phylogenetic analysis indicated that the lineage leading to *Trapa* was diverged from the lineage to *Sonneratia* approximately 23 million years ago. These two assemblies provide valuable resources for future evolutionary and functional genomic research and molecular breeding of water chestnut.

## Background & Summary

*Trapa* L., known as water chestnut or water caltrop, is the only genus of Trapaceae. Although the Angiosperm Phylogeny Group (APG) IV treated Trapaceae belonging to Lythraceae, the term “Trapaceae” is still used by some scholars today due to a handful of morphological differences between the two families^1^. *Trapa* plants are annual floating-leaved herbs naturally growing in temperate, subtropical and tropical regions of the Old World, and invasive in Australia and North America^2^. They reproduce sexually and/or asexually and have a high degree of autogamy^3,4^. The genus has two diversity centers, i.e. the Yangtze River Basin (central China) and the Amur River- Tumen River Basin (the border between China and Russia)^5^. *Trapa* plants have high edible value because of their large starchy seeds, which has a long history of consumption. In China, archaeological studies found that water chestnut was widely eaten during the Neolithic Age (7000-2000 BC) with 21 unearthed sites in the basins of the Yellow River and Yangtze River^6^. In ancient Europe, inhabitants also gathered water chestnut seeds as part of their diet between 4000 and 1000 BC^7^. The cultivation of water chestnut can be traced back to the Tang (618–907 AD) and Song (916–1279 AD) dynasties^8^ in the middle and lower reaches of the Yangtze River. At present, it is an important aquatic crop widely grown in China and India^9^. Additionally, the tender *Trapa* seeds, stems and leaves are used as vegetables because of the fresh and sweet taste, whereas their seed pericarps are traditional Chinese medicine because of their bioactive components in the treatment of cancer, inflammation and atherosclerosis^10–12^. Furthermore, *Trapa* has significant ecological value in improving water quality due to its strong absorption capacity for heavy metals and pollutants^13^.

A better understanding of species identification, evolutionary relationships and genetic information will greatly facilitate the effective management and sustainable utilization of wild plant resources. However, the classification of *Trapa* species is still open to debate because of their similar morphology of vegetative organs and the highly variable seeds. Some scholars argued that the genus contained more than 20, 30 or 70 species, while others merged them into one or two polymorphic species^14^. The quantitative taxonomic studies based on morphological variations showed that *Trapa* species with similar seed sizes were closely related, and all species were divided into two branches, the large- and small-seed clusters^15^. This was well supported by the molecular studies based on chloroplast (cp) sequences^14,16^. The cp genome analysis also showed that both the geographical origin and tubercle morphology of seeds were of great significance for deducing relationship within *Trapa*^14^. Cytological studies showed two different chromomeric numbers in *Trapa* (2n = 2x = 48 and 2n = 4x = 96) and suggested that the tetraploid might be a hybrid of diploids^17^, which was supported by molecular analyses based on allozymes as well as nuclear and chloroplast DNA sequeences^18,19^. The existence of the two distinct subgenomes was directly confirmed by the recently published chromosome-level assembly of a tetraploid *Trapa natans* (AABB) genome^8^. Furthermore, the resequencing data exhibited that large-seed species contained both diploids (2n = 2x = 48, AA) and tetraploids (2n = 4x = 96, AABB), and the small-seed ones only contained diploids (2n = 2x = 48, BB)^8^. It is a pity that the genome sequences of representatives of the ‘AA’ and ‘BB’ genomes are not available, though such species are very common in the *Trapa* genus.

Here, we sequenced the genomes of the typical cultivated species *Trapa bicornis* Osbeck (AA) and a small-seed species *Trapa incisa* Sieb. et Zucc. (BB), which would greatly deepen the understanding of *Trapa* diversity and the origin of tetraploid *Trapa*. *De novo* assembly using PacBio high-fidelity (HiFi) long reads generated 479.90 and 463.97 Mb contigs for *T. bicornis* and *T. incisa* with N50 values of 13.51 and 13.77 Mb, respectively. After scaffolding by Hi-C reads, 98.0% and 98.1% of the contigs could be successfully anchored into 24 pseudo-chromosomes for each genome, respectively. We predicted 33,306 and 33,315 protein-coding genes in *T. bicornis* and *T. incisa* genomes, respectively. Despite good collinearity, there were 159,232 structural variations (SVs) identified between the genomes of *T. bicornis* and *T. incisa*, overlapping with more than 11 thousand genes. Divergence time estimation indicated that *T. bicornis* and *T. incisa* diverged around 1.51 million years ago. The generation of the two genomes provides baseline information of the diversity of *Trapa* species, which will eventually facilitate functional genomic analysis and molecular breeding of water chestnut.

## Methods

### Sample collection and sequencing

**Fig. 1:**
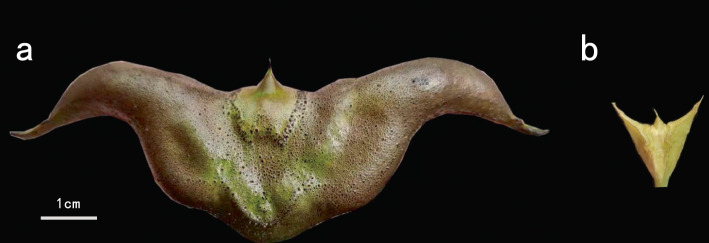
The seeds of *T. bicornis* Osbeck var. *bicornis* (**a**) and *T. incisa* Sieb. & Zucc. var. *incisa* (**b**).

Seeds of *T. bicornis* and *T. incisa* were collected from Honghu (29.39°N/113.07°E), Hubei province, China (Fig. 1). Plants were cultured outdoors from March to July in water tanks in Wuhan Botanical Garden, Chinese Academy of Science, Hubei province, China. The 90-day-old individuals for each species were used for the DNA/RNA extractions.

Genomic DNA was isolated from fresh young leaves using Cetyltrimethylammonium bromide (CTAB) method^20^. A total amount of 1.5 µg DNA per sample was used as input material for the Illumina paired-end library construction. Each library with an average insert size of 350 bp was generated using Truseq Nano DNA HT Sample preparation Kit (Illumina USA) following manufacturer’s instructions. These libraries were sequenced by Illumina HiSeq X Ten system. A total of 125.97 Gb and 53.14 Gb paired-end reads (PE150) covering roughly 183.38 × and 112.42 × of genomes were generated for *T. bicornis* and *T. incisa*, respectively (Table 1).

**Table 1:** Sequencing data of *T. bicornis* and *T. incisa* genome.

Species	Library type	Raw data (Gb)	Clean data (Gb)	Read N50/ length (bp)*	Coverage (×)
*T. bicornis*	**Illumina**	125.97	125.21	150	183.38
	**PacBio HiFi**		24.11	17,588	49.23
	**Hi-C**	111.79	111.06	150	228.31
	**RNA**	34.05	30.87	150	
*T. incisa*	**Illumina**	53.14	52.82	150	112.42
	**PacBio HiFi**		20.42	13,963	43.20
	**Hi-C**	103.65	102.55	150	219.26
	**RNA**	36.68	35.21	150	

* For PacBio Hifi, this number is read N50; for others, this number is read length.

For PacBio long-read sequencing, about 10 µg genomic DNA were sheared into fragments of 10-20 kb in length by g-TUBE (Covaris USA). The fragmented DNA was purified by AMPure PB magnetic beads. The High-fidelity (HiFi) libraries were generated using SMRTbell Express Template Prep Kit 2.0 and sequenced on PacBio Sequel IIe platform (Pacific Biosciences, Menlo Park, USA). A total of 24.11 Gb and 20.42 Gb HiFi reads with N50 sizes of 17,588 bp and 13,963 bp were obtained using the CCS (Circular Consensus Sequencing) software with default parameters (https://ccs.how/), which covered 49.23 × and 43.20 × of *T. bicornis* and *T. incisa* genomes, respectively (Table 1).

The high-throughput chromosome conformation capture (Hi-C) libraries were constructed using 5 µg DNA. The DNA crosslinking was performed by 4% formaldehyde. The linked DNA was digested with DpnII restriction endonuclease, labelled with biotin-14-DCTP and then ligated by T4 DNA Ligase. The ligated DNA was sheared into 200-600 bp fragments and sequenced on Illumina HiSeq X Ten system with the paired-end module. About 111.79 Gb and 103.65 Gb of raw data were obtained for *T. bicornis* and *T. incisa*, respectively (Table 1).

RNA was extracted from roots, petioles, leaves, flowers and fruits, respectively, using Tiangen RNAprep pure plant kit (Tiangen Biotech, China). Libraries were constructed using NEBNext UltraTM RNA Library Prep Kit (NEB, USA) according to the manufacturer’s instructions, and sequenced on Illumina Novaseq. 6000 platform. RNA-seq datasets from different tissues of the same species were combined as evidence for genome annotation. A total of 34.05 Gb and 36.68 Gb RNA-seq reads were obtained for *T. bicornis* and *T. incisa*, respectively (Table 1).

### Genome assembly

**Fig. 2:**
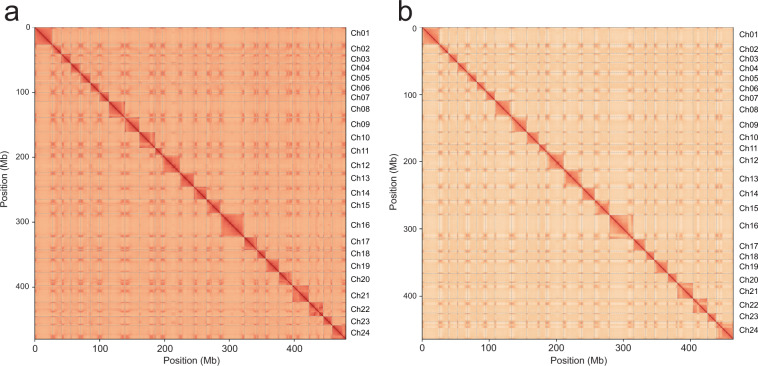
Hi-C interactions among the 24 pseudo-chromosomes of *T. bicornis* (**a**) and *T. incisa* (**b**) genomes. Weak to strong interactions are shown in yellow to red.

The PacBio HiFi reads of each genome were *de novo* assembled by using hifiasm v0.16.1^21^ with default parameters. The assemblies had a total size of 489.65 Mb and 472.74 Mb, containing 325 and 262 contigs with N50 sizes of 13.52 Mb and 13.77 Mb for *T. bicornis* and *T. incisa*, respectively (Table 2). The cleaned Hi-C reads were mapped to the corresponding contigs using Juicer v1.9.9^22^. The unique mapped reads were taken as input for 3D-DNA pipeline v180114^23^ with parameters “-r 0” and then sorted and corrected manually by using JuicerBox v1.11.08^24^. Finally, a total of 24 pseudo-chromosomes was obtained, which contained 98.01% and 98.14% of the assembled contigs for *T. bicornis* and *T. incisa*, respectively (Fig. 2).

**Table 2:** Assessment of *T. bicornis* and *T. incisa* assemblies.

	*T. bicornis*	*T. incisa*
**Contig level**		
Assembly length (bp)	489,648,690	472,743,997
Longest contig (bp)	20,804,803	25,982,365
Number of contigs	325	262
Mean contig length (bp)	1,506,611	1,804,366
Contig N50 (bp)	13,515,041	13,768,160
N50 contig number	15	14
GC content	38.33%	38.16%
Merqury (QV)	49.7	43.91
PE reads mapping rate	99.88%	99.61%
Genome covered by at least 5 reads	99.68%	99.71%
BUSCO	97.70%	97.60%
**Chromosome level**		
Anchor ratio	98.01%	98.14%
Chromosome length (bp)	479,895,984	463,973,675
Scaffold N50 (bp)	21,554,504	21,690,287

We assessed the integrity of the genomes using the BUSCO v5.0 (Benchmarking Universal Single-Copy Orthologs)^25^ with the ‘embryophyta_odb10’ database. The *T. bicornis* and *T. incisa* assemblies contained 97.70% [S:85.10%, D:12.60%, F:0.90%, M:1.40%, n:1614] and 97.80% [S:84.70%, D:13.10%, F:0.80%, M:1.40%, n:1614] of the 1,614 conserved genes, respectively, which are similar to the corresponding values of the diploid *T. natans* (C: 96.41% [S: 84.76%, D: 11.65%, F: 0.43%, M: 3.16%, n: 1614])^26^. Based on the Illumina PE150 reads, we assessed the consensus quality values (QV) of the two assemblies using Merqury v2020-01-29^27^ with “k-mer = 20”. For *T. bicornis* and *T. incisa* assemblies, the mapping rate of the reads were 99.88% and 99.61%, respectively, and the QV values were 49.70 and 43.91, respectively (Table 2). These evaluations indicated that the two genome assemblies were of considerable completeness, contiguity and accuracy.

### Genome annotation

Custom repeat libraries for each genome were constructed by screening the genome using LTR_finder^28^, ltrharvest^29^ and RepeatModeler-2.0.2a^30^. Then, the non-redundant repeats from Repbase^31^ and Dfam^32^ databases were extracted and added to the custom libraries. RepeatMasker v 4.1.2-p1 (http://www.repeatmasker.org) was used to identify repeat sequences based on the custom libraries. A total of 307.95 Mb (62.88%) and 295.42 Mb (62.49%) repetitive sequences were annotated in the *T. bicornis* and *T. incisa* genomes, respectively (Table 3).

**Table 3:** Genome annotation of repetitive sequences and protein-coding genes.

	*T. bicornis*	*T. incisa*
**Repetitive sequence**		
Total repeative sequence	62.88%	62.49%
Retroelements	39.50%	41.17%
LTR-Ty1/Copia	3.93%	2.65%
LTR-Gypsy/DIRS1	29.69%	32.06%
DNA transposons	4.55%	5.26%
**Protein-coding gene**		
Gene number	33,306	33,315
Mean gene length (bp)	2,522.59	2,493.22
Mean CDS length (bp)	228.33	228.5
CDS number per mRNA	5.48	5.45
BUSCO	97.70%	98.10%
single-copy BUSCOs (%)	85.20%	85.00%
duplicated BUSCOs (%)	12.50%	13.10%
**Functional annotation**		
SwissProt	31,172 (93.59%)	31,213 (93.69%)
NR	31,193 (93.66%)	31,229 (93.74%)
TrEMBL	25,037 (75.17%)	25,039 (75.16%)
KEGG	28,097 (84.36%)	28,126 (84.42%)
InterPro	30,959 (92.95%)	30,945 (92.89%)
GO	21,159 (63.53%)	21,138 (63.45%)
Total	31,360 (94.16%)	31,406 (94.27%)

For protein-coding gene annotation, we employed RNA-seq-based, *ab initio* and homologue-based predictions to identify gene models. The clean RNA-seq reads were aligned to the assemblies using HISAT2 v2.2.1^33^, and then the alignment was converted to gtf format by StringTie2 v2.1.6^34^. Furthermore, TransDecoder v5.5.0^35^ was used to identify the open reading frame (ORF) and modify the boundaries of exons. The *ab initio* gene predictions were generated by three *de novo* predicting programs, including Augustus-3.3.3^36^, SNAP v2006-07-28^37^ and GlimmerHMM 3.0.4^38,39^. Proteins from *Punica granatum*^40^, *Arabidopsis thaliana* TAIR10^41^, *Eucalyptus grandis*^42^, *Melaleuca alternifolia*^43^ and tetraploid *Trapa natans*^8^ were aligned to the genomes using TBLASTN^44^. The homologous genes were identified using Exonerate v2.2.0^45^. The RNA-seq evidences, *ab initio* predictions and homolog evidences were fed to MAKER v3.01^46^ to generate the final gene set. A total of 33,306 and 33,315 protein-coding genes were predicted in the *T. bicornis* and *T. incisa* genomes, respectively.

Functional annotation of protein-coding genes were evaluated based on five public databases, including GO (http://geneontology.org/), KEGG (https://www.kegg.jp/), GenBank nr (https://www.ncbi.nlm.nih.gov/), Uniprot (https://www.uniprot.org/) and Interpro (http://www.ebi.ac.uk/interpro/), using DIAMOND v2.0.13.151^47^. A total of 31,360 (94.14%) and 31,406 (94.27%) genes were successfully annotated in at least one database for *T. bicornis* and *T. incisa*, respectively (Table 3). The BUSCO completeness values were 97.70% and 98.10% of the predicted proteins of *T. bicornis* and *T. incisa*, respectively (Table 3).

### Variations between the *T. bicornis* and *T. incisa* genomes

**Fig. 3:**
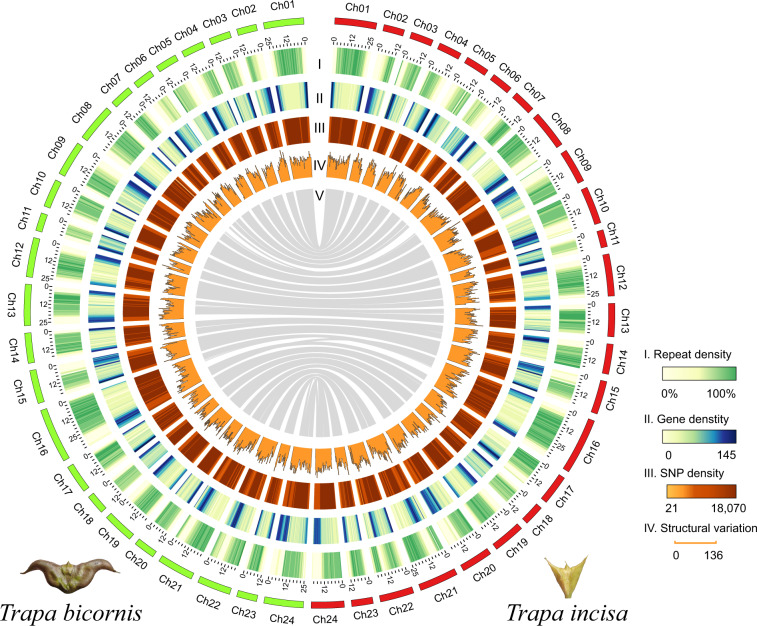
Genomic landscape of *T. bicornis* and *T. incisa*. Window size is 500 kb. The cycles from outer to inner show (I) densities of repetitive sequences, (II) gene, (III) SNP and (IV) SV numbers in sliding windows. All statistics were normalized by log scale.

Single nucleotide polymorphisms (SNPs) between the genomes of *T. bicornis* and *T. incisa* were detected by alignment of the two assemblies using NUCmer from MUMMER4^48^. We set the minimum alignment length to 100 bp and retained the uniquely matching fragments. A total of 9,449,234 SNPs were identified by show-snps tool from MUMMER4^48^ (Fig. 3).

To identify SVs, *T. incisa* genome was mapped to *T. bicornis* genome by using Minimap2^49^ with the parameter “-ax asm5”. Assemblytics was adopted to extract unique alignments and identify SVs based on them^50^. Protein-coding genes overlapping with SV regions were retrieved by BEDTools v2.29.1^51^. The final SVs were classified into seven categories: deletion, insertion, repeat contraction, repeat expansion, tandem contraction, tandem expansion and substitution. A total of 159,232 SVs were identified between *T. bicornis* and *T. incisa* genomes, which accounted for 110.49 Mb and 140.13 Mb sequences of the two genomes, respectively (Table 4). These SVs overlapped with 11,265 and 11,621 genes of the two *Trapa* genomes, respectively.

**Table 4:** The structure variations detected between the *T. bicornis* and *T. incisa* genomes.

SV type^*^	Number	SV size in *T. bicornis* (bp)	SV size in *T. incisa* (bp)
Deletion	65,705	16,222,557	7,853
Insertion	63,465	8,530	15,736,483
Substitution	9,225	7,715,182	7,709,820
Repeat contraction	9,888	62,795,688	16,706,904
Repeat expansion	10,600	22,711,493	99,133,601
Tandem contraction	131	649,261	78,849
Tandem expansion	218	388,310	756,762
Total	159,232	110,491,021	140,130,272

* The SV type indicates the variation detected in *T. incisa* genome relative to *T. bicornis* genome.

### The synteny between the published tetraploid *T. natans* genome and the present two diploid *Trapa* genomes

**Fig. 4:**
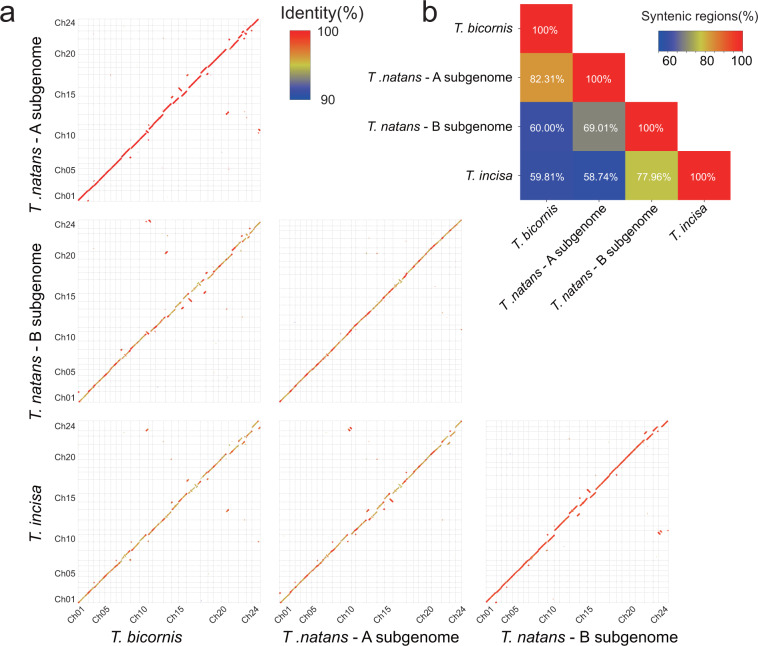
Synteny between genomes of *T. bicornis*, *T. incisa* and subgenomes of tetraploid *T. natans*. (**a**) Pairwise comparisons of the genomes of *T. bicornis*, *T. incisa* and the two subgenomes of tetraploid *T. natans*. (**b**) The percentages of syntenic regions of each comparison.

Our new assemblies provided great resource for investigating the origin of the *Trapa* tetraploid and the genomic changes post-polyploidization. The genomes of *T. bicornis* and *T. incisa* and the two subgenomes of the published tetraploid genome were pairwise aligned with each other by using MUMMER4^48^ (Fig. 4). The syntenic regions were extracted from the alignments with the software syri-1.4^52^. Clearly, the *T. bicornis* and *T. incisa* genomes possessed the highest percentage of syntenic regions with the A and B subgenomes of *T. natans*, respectively, suggesting that the formers represented the ancestry genomes of the latter two, separately. The percentage of syntenic regions between the A and B subgenomes (69.01%) was higher than that between the *T. bicornis* and *T. incisa* genomes (59.81%), evidencing homoeologous recombination events after tetraploidization^53^.

### Comparative genomics and divergence time estimation

**Fig. 5:**
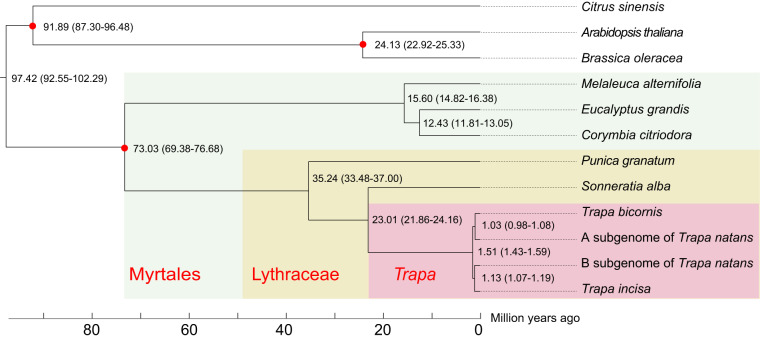
Phylogenetic tree with estimated divergence times. The maximum likelihood tree was constructed based on 1,106 single-copy orthologous genes. The red dots at the nodes indicated that the values were supported by fossil evidence.

Using OrthoFinder v2.5.2^54^, orthologous groups were constructed for 11 species, including *Arabidopsis thaliana*^41^, *Brassica oleracea*^55^, *Citrus sinensis*^56^, *Corymbia citriodora*^26^, *Eucalyptus grandis*^42^, *Melaleuca alternifolia*^43^, *Punica granatum*^40^, *Sonneratia alba*^57^, *Trapa bicornis*, *Trapa incisa* and tetraploid *Trapa natans*^8^ (AABB), which was divided into two subgenomes. A total of 1,105 single copy orthologues were obtained, and they were aligned using MUSCLE v3.8.31^58^. The alignments of protein sequence were converted into nucleotide sequences. The final alignments of orthologous groups were concatenated to build a maximum likelihood phylogenetic tree using RAxML-8.2.12^59^ with “GTRGAMMA” model. The figure of phylogenetic tree was visualized by iTOLv6^60^. Divergence times among the species were estimated using the MCMC tree program implemented in PAML v4.9i^61^. The reference divergence time was obtained from http://timetree.org/. The three species (*Citrus sinensis*, *Arabidopsis thaliana* and *Brassica oleracea*) were constrained as root in the time-calibrated phylogeny. Due to the lack of strong morphological evidence, the relationship between *Trapa* and Lythraceae has been unclear historically^62^. Here, our phylogenetic tree (Fig. 5) showed that *Trapa* was sister to the genus *Sonneratia* (Lythraceae s.l.), which was also supported by previous studies based on chloroplast and nuclear sequences^14,63,64^. According to the time-calibrated phylogeny, the *Trapa*-*Sonneratia* clade diverged from *Punica* (Lythraceae) at ca 35.24 million years ago. Then, the two genera (*Trapa* and *Sonneratia*) diverged ca 23 Mya ago, and the two *Trapa* species with distinct genomes (*T. bicornis*: AA; *T. incisa*: BB) diverged ca 1.5 Mya.

## Data Records

The raw data of Illumina PE150 reads, PacBio HiFi long reads and Hi-C reads from *T. bicornis* were submitted to the National Center for Biotechnology Information (NCBI) SRA (Sequence Read Archive) database with accession number SRR22185068^65^, SRR22185067^66^, SRR22185066^67^ under BioProject accession number PRJNA893431^68^. The RNA-seq data for the five tissues are also under the PRJNA893431^68^. For *T. incisa*, the raw data of Illumina, PacBio and Hi-C sequencing had been deposited in SRA database as SRR22094614^69^, SRR22094613^70^ and SRR22094612^71^ under PRJNA894094^72^. And the RNA-seq data are also under the same BioProject accession. The assembly genome files were stored in GenBank database under the accession GCA_030064425.1^73^ and GCA_030064435.1^74^, respectively. The genomes and annotation files and raw sequencing data have also been uploaded in National Genomics Data Center (NGDC) under PRJCA012133^75^ and PRJCA012134^76^.

## Technical Validation

The quality scores across all bases and GC content of the Illumina raw sequencing data were inspected by FastQC v0.11.9 (https://www.bioinformatics.babraham.ac.uk/projects/fastqc/). Contig level and chromosome level of the assemblies were assessed in four ways: N50 for continuity, QV for accuracy, BUSCO for completeness and paired-end reads mapping rate for consistency with raw data. The protein-coding genes were verified by values of BUSCO and functional databases annotation. For construction of phylogenetic tree, each branch received 100% bootstrap values.

## Data Availability

The scripts and command lines were uploaded on the github (https://github.com/fcbayern31/A-pipeline-for-common-genomic-analysis.git). All softwares, which are in the public domain, were used in accordance with the official instructions. Anything not specified in the method is executed with default parameters.
